# Digital Twins in Industry 5.0

**DOI:** 10.34133/research.0071

**Published:** 2023-03-14

**Authors:** Zhihan Lv

**Affiliations:** Department of Game Design, Faculty of Arts, Uppsala University, Uppsala, Sweden.

## Abstract

This work aims to explore the impact of Digital Twins Technology on industrial manufacturing in the context of Industry 5.0. A computer is used to search the Web of Science database to summarize the Digital Twins in Industry 5.0. First, the background and system architecture of Industry 5.0 are introduced. Then, the potential applications and key modeling technologies in Industry 5.0 are discussd. It is found that equipment is the infrastructure of industrial scenarios, and the embedded intelligent upgrade for equipment is a Digital Twins primary condition. At the same time, Digital Twins can provide automated real-time process analysis between connected machines and data sources, speeding up error detection and correction. In addition, Digital Twins can bring obvious efficiency improvements and cost reductions to industrial manufacturing. Digital Twins reflects its potential application value and subsequent potential value in Industry 5.0 through the prospect. It is hoped that this relatively systematic overview can provide technical reference for the intelligent development of industrial manufacturing and the improvement of the efficiency of the entire business process in the Industrial X.0 era.

## Introduction

The manufacturing industry has transformed rapidly, and manufacturers worldwide face the challenge of improving productivity. Germany introduced Industry 4.0 in 2011 to improve its industrial competitiveness [1–3]. Industry 4.0 is founded on the division of industrial development phases. Initially, Industry 1.0 announced the epoch of the steam engine. Industry 2.0 is a revolution of electricity. Industry 3.0 was born as the information age, and Industry 4.0 was the age of intelligence that used information technology to promote industrial upgrades. The Fifth Industrial Revolution (Industry 5.0 or 5IR) is proposed by the European Union in 2021 as the Internet and artificial intelligence (AI) era in industrial design and manufacturing. It considers people orientation, sustainability, and flexibility [4,5]. Xu et al. [6] revealed that Industry 4.0 is considered technology-driven, while 5IR is value-driven. Mourtzis [7] believed that the focus of 5IR should be on the design and development of a suitable framework to achieve process optimization based on semantic integration through the effective use of big data. 5IR is still an industrial vision for developed countries because of limited efficiency and productivity. In particular, it strengthens the role and contribution of industry to society. 5IR is still a future orientation for most enterprises, where the potential of the Industry 4.0 era must be fully tapped [8]. Thereby, it helps realize the leap from technology to leadership, coordinate resources and cross functions, handle the operational intelligence data provided by digitalization, and be more predictable to work units and factories.

Emerging technologies are applied in modern industrial production [9]. Digital Twins (DT) Technology fully uses data such as physical models, sensor updates, and operation history to integrate multidisciplinary, multiphysical, multiscale, and multiprobability simulation processes. DT can collect various physical models' information through simulation technology and map a digital virtual twin of the real entities [10–12]. In this sense, DT can monitor digital entities and operating indicators in real time. It projects the natural world through data accumulation and AI and feeds back the results to the real world. Statistics show that 85% of Internet of Things (IoT)-native devices use DT to safeguard Information Security. Therefore, DT-powered smart city construction has become a research hotspot [13,14]. In urban construction, analyzing infrastructure is the hardcore in the IoT. The existing construction management fails to consider the dynamic needs of times and society. Hence, implementing a DT-powered analysis model can promote the infrastructure industry and has a high theoretical value.

DT originated in industrial manufacturing [15]. Židek et al. [16] believed that DT could visualize the real state of manufacturing system as 3-dimensional (3D) simulation with real-time implementation. In product research and development, DT can virtually model the product that can be verified through simulation experiments. For manufacturing, DT could simulate equipment operation and parameter adjustment. Also, DT could improve the products' reliability and availability and reduce development and manufacturing risks [17,18]. DT was crucial in the maintenance phase. For example, continuously collecting and analyzing operation data could predict the best time point for maintenance. The reference basis of the maintenance cycle could be offered [19,20]. Meanwhile, DT served as the reference for fault points and probability. DT has substantial enhanced benefits and reduced costs in industrial manufacturing, attracting industrial tycoons in a special field [21–23].

At present, the relevant research on the application of DT in the industrial field is mainly used for debugging and experimenting in the virtual space [24,25] and ultimately achieves the best operation effect of the machine and other related content. However, the overall development process and summary analysis of the application of DT in industrial manufacturing are relatively rare. To this end, this work conducts a comprehensive research on the DT in 5IR. The specific review method is as follows. A computer is used to retrieve the core set of the Web of Science database. Digital Twins, Industry 4.0, Industry 5.0, Man-Machine Integration, Virtual Reality Modeling (VRM), etc. are selected as the keywords for retrieval. The search results were 1,921 articles. After screening and eliminating invalid and duplicate data, a total of 1,435 English articles of relevant research in the context of 5IR from 2013 to 2022 were finally determined. After further analysis of the title, abstract, and full text, 136 relevant documents were finally selected.

In this review study, the overall organizational structure is as follows. 5IR Architecture and Its VRM Technology introduces the background and architecture of 5IR and compares the additional features of 5IR with previous literature on industrial evolution. On this basis, a review discusses the literature related to potential applications in 5IR and key modeling techniques. Application Values of DT in 5IR VRM discusses the relevant literature on the application of DT, an emerging technology, in VRM of 5IR and analyzes the implementation process of key technologies. Discussion and Prospect looks forward to the potential application value of DT in 5IR and the subsequent research direction. Conclusion summarizes the review results to provide technical reference and reference for the intelligent development of the industrial field.

## 5IR Architecture and Its VRM Technology

### Evolution motivation of Industry 4.0 to 5IR

**Fig. 1.**
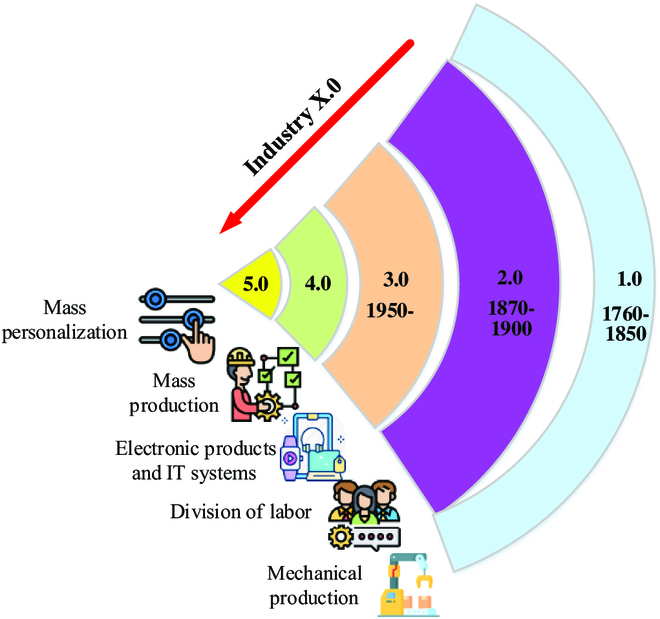
Industrial Revolution from Industry 1.0 to Industry 5.0 (inspired by [34]). IT, information technology.

Until the 18th century, the world entered Industry 1.0, involving textile, steam power, steel, cement, chemical, natural gas, glass, paper, mining, agriculture, and transportation. The industrial revolution's achievement can be seen in agriculture, transportation, and sustained economic growth. Industry 2.0 began in the 19th century. It focused on steel, railway, electrification, machine tools, paper, petrochemical, maritime technology, rubber, automobile, fertilizer, engine, turbines, telecommunications, and modern business management. At this stage, separating parts production and product assembly has created a new and efficient mode of mass production. Industry 3.0 saw the popularization of electronic and information technologies. The extensive application of electronic and information technology has improved the automatic control of the manufacturing process. Industry 4.0 was developed with the concept of intelligent manufacturing in 2011. Mainly, it aims to maximize productivity through labor-intensive large-scale production using emerging technologies [26]. Sharma and Villányi [27] proposed an end-to-end 2-way authenticated secure data transmission technology in the context of Industry 4.0. They found that the technology ensures the integrity and stability of the entire system during the scale-out phenomenon. Besides, the technique has the lowest communication overhead, computational cost, and round-trip time. Javaid et al. [28] found that Industry 4.0 increases production flexibility and enables factories to respond quickly to market changes. In addition, the factory control system automatically changes output according to changing utilization rates, thereby reducing production costs. Javaid and Haleem [29] reasoned that 5IR was the future evolution direction of the industrial field, aiming to use human creativity to collaborate with efficient and intelligent machines. Figure 1 shows the revolution from Industry 1.0 to Industry 5.0.

The 21st century has witnessed the fast development of state-of-the-art information technologies such as IoT, cloud computing, big data technology (BDT), mobile Internet, and AI. Mourtzis and Doukas [30] illustrated the evolution of manufacturing paradigms, their underlying principles, and the linkages between them, taking into account each era's remarkable technological advances and other sociopolitical reasons. When Germany proposed industry 4.0 in 2011 [31,32], the progress of information technology was strengthened alongside the highly transformative impact brought by digital, data-driven, and interconnected industries. Krugh and Mears [33] claimed that Industry 4.0 used the cyber-physical system (CPS) to digitize the production supply, manufacturing, and sales. As such, it achieved rapid, effective, and personalized product supply. Essentially, it fully used CPS to push the manufacturing industry to digital and intelligent transformation. Industry 4.0 was believed to have the features of deep integration of the digital and physical worlds. Industry 4.0 provided guiding principles for European industrial innovation, technological development, and digital transformation, which has been recognized by the world's major economies [34–36]. Sader et al. [37] believed that Industry 4.0 could realize the interconnection of machines and make the manufacturing Industry intelligent through mutual control of equipment throughout the life cycle. Industry 4.0 prioritized process automation, minimizing human intervention in the manufacturing process.

Industry 4.0 stresses system autonomy and raises questions. For example, what role should human intelligence play? After machines replace people, how should the employment problem be solved? Mourtzis et al. [38] regarded 5IR as a framework to promote the coexistence of industry and emerging social trends and needs and provide a basis for promoting the transition from Industry 4.0 to Society 5.0. Liao et al. [39] proposed a DT model for cloud-edge device collaboration reliability and communication efficiency for low-carbon power device management. After experiments, they found that it performed well in terms of loss function, communication efficiency, and carbon emission reduction. Zhou et al. [40] presented a federated learning-based DT framework and a safe and efficient DT-assisted resource scheduling algorithm. It was found that the model has excellent performance in terms of cumulative iteration delay, DT loss function, energy consumption, and lack of access priority in the context of 5G edge computing authorization. Zemtsov et al. [41] mentioned that the industry could exclude industrial workers from employment while promoting economic prosperity. Otherwise, many social problems might arise. Nor should it cause great pollution and damage to the environment to affect the health of industrial workers and the living environment of human beings. Therefore, the decision-makers, technical experts, and policy makers discussed 5IR to systematically solve the status of people and the environment in industrial production. The current 5IR concept utilizes the unique creativity of human experts to collaborate with powerful, intelligent, and accurate machines [42]. Leng et al. [43] reviewed the development of 5IR and the 3 main features of 5IR: people-oriented, sustainability, and flexibility. They also constructed a 3D system that realizes the technical, realistic, and application dimensions of 5IR structure. Finally, they discussed the key enablers of 5IR, future implementation paths, potential applications, and challenges of real-world scenarios. It is expected that 5IR will combine high-speed and accurate machines with human critical cognitive thinking. Users can choose personalized and customized products according to their tastes and needs. Kaasinen et al. [44] thought that 5IR would significantly improve manufacturing efficiency and create multifunctional connections between humans and machines. It could realize human–machine interaction and continuous monitoring. 5IR improves production quality by offloading mechanical tasks with high computational complexity to robots/machines and introducing human ideas into critical thinking assignments.

### Connotation and system architecture of 5IR

In April 2021, the European Union put forward the 5IR plan with the objectives of sustainable development, people orientation, and flexible economy. It is believed to optimize Japan's Social 5.0 and Germany's Industry 4.0. The 10-year development of Industry 4.0 no longer pays attention to the original principles of social equity and sustainability. Instead, it emphasizes digital and AI-driven technologies to improve production scalability and efficiency. Bryndin [45] observed that 5IR offered a different perspective and highlighted deep learning and innovative capability in supporting long-term services to humanity globally. 5IR, a developing paradigm, has triggered a new industrial revolution. 5IR must comply with internationally recognized standards for full acceptance and high standards. Huang et al. [46] found that 5IR was a people-centered solution. Cooperative robots would be an integral part of it and give a typical application scenario based on cooperative robots. In this application scenario, the cooperative robot could perceive the position and state of the person through visual means, such as cameras, and predict and understand the person's intention by watching and learning. When people performed manual operations, the robots could learn to prepare for the next operation. Maddikunta et al. [47] concluded that human beings had great potential and should fully exert their creativity, agility, and other abilities in the industrial system as an essential production factor. In addition, the industry should serve people, the main body of industrial creation. Aslam et al. [48] inferred that 5IR shifted the center of industrial development back to people. It facilitated people's living environment, working environment, personal privacy, and social value expression. This was far more than productivity improvement and economic growth. Moreover, sustainable development, people orientation, and flexibility were the key concerns of 5IR.

In terms of technology, Fraga-Lamas et al. [49] revealed that 5IR seized the promise of advanced digital development, BDT, and AI. At the same time, it emphasized the role of these technologies in meeting new and urgent needs in industry, society, and the environmental landscape. Thus, data and AI-optimized production flexibility made the value chain more stable. New technologies could achieve recycling and sustainability. Akundi et al. [50] discovered that the key technologies of 5IR were people-oriented solutions and human–computer interaction (HCI). They connected the advantages of people and machines. The whole system was modeled on the basis of the real-time DT and analog system. On the other hand, Zizic et al. [51] noted that the network security data transmission, storage, and analysis technology were the basics of 5IR. They could handle data and system interoperability. Huang et al. [46] thought that the key to 5IR was to model the whole industrial system on the basis of the real-time DT and analog system. According to the above literature review, Table 1 summarizes the definitions of 5IR.

**Table 1.** Relevant definitions of the connotation of 5IR.

Authors	Thesis title	Core point
Bryndin [45]	Formation and management of 5IR by systems with artificial intelligence and technological singularity	Support industry to provide long-term services for humanity globally
Huang et al. [46]	5IR and Society 5.0—Comparison, complementation, and co-evolution	People-centric solutions
Maddikunta et al. [47]	5IR: A survey on enabling technologies and potential applications	Human beings were a critical factor in production.
Aslam et al. [48]	Innovation in the era of IoT and 5IR: Absolute innovation management (AIM) framework	The goal of industrial development was people-centered.
Fraga-Lamas et al. [49]	Green IoT and edge AI as key technological enablers for a sustainable digital transition towards a smart circular economy: An 5IR use case	Recycling and sustainability through technology
Akundi et al. [50]	State of 5IR—Analysis and Identification of Current Research Trends	Connecting the advantages of man and machine
Zizic et al. [51]	From Industry 4.0 towards 5IR: A Review and Analysis of Paradigm Shift for the People, Organization, and Technology	Network security data transmission, storage, and analysis
Huang et al. [46]	5IR and Society 5.0—Comparison, complementation, and co-evolution	Real-time DT and analog system

**Fig. 2.**
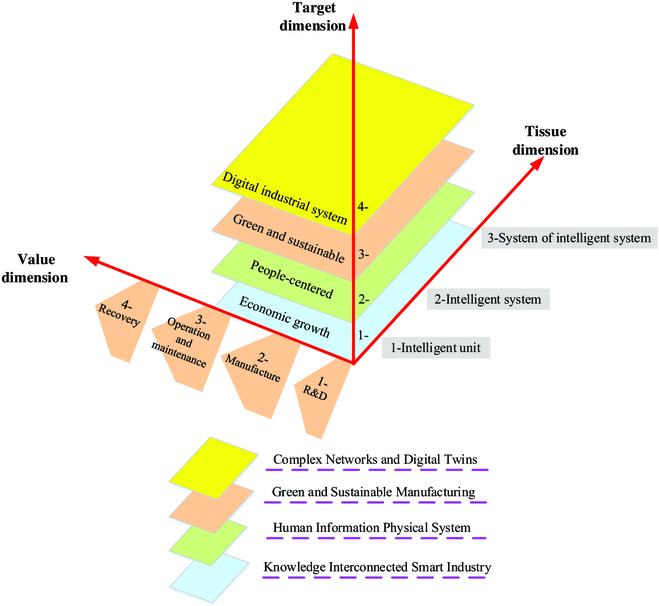
Architecture of 5IR (inspired by [50]). R&D, research and development.

Following the literature overview, this work summarizes the connotation of 5IR. 5IR enables production to respect the boundaries of the earth and the interaction, coordination, and integration of people, machines, and things at the knowledge level. As such, industrial workers and their interests are placed at the core of production to achieve different social goals and economic growth. It can provide prosperity steadily and sustainably. 5IR reflects the power of industry in achieving social goals other than employment and growth by respecting the earth's ecology and putting the welfare of industrial workers at the center of the production process. It is the cornerstone of stability and prosperity [52–54]. Figure 2 shows the architecture of 5IR.

According to the 5IR architecture in Fig. 2, human creativity and rapid response to uncertain situations are a crucial part of the industrial production process and should be fully explored to improve product innovation and quality efficiency.

### Man–machine integration in 5IR era

Industry 4.0 integrates the physical and the virtual world through CPS and connects people, machines, and equipment over the IoT. Zong et al. [55] noted that the physical-virtual crossing had realized the horizontal and vertical interconnection of the entire value chain. It formed a new value network and ecosystem from customers to suppliers across the entire product life cycle and different functional departments. Value-added creation was more efficient, personalized, higher-quality, service-oriented, more traceable, and more flexible. At the same time, it will be connected to production in different ways to form an integration chain covering the whole life cycle. 5IR will benefit the economy, ecology, and society, the triple bottom line of sustainable development. Mehdiabadi et al. [56] believed that, like Industry 4.0, 5IR would rely on data, equipment, and AI to coordinate commerce and trade. These components all depended on memory, similar to the human brain's functioning. In fact, memory placed intelligence in AI, providing data to run algorithms and the context of actions and reactions.

An ideal picture in the 5IR era is that operators and robots can cooperate in communication on the same workbench, just like experienced partners. Traditional industrial robots have no "soul". They can guarantee quality and quantity, higher accuracy, and greater strength than human beings and can work 24 hours without interruption. However, they could only perform repetitive actions in a fixed environment [57]. Tyagi and Sreenath [58] believed that the CPS used computing, communication, and control to manufacture new technologies or next-generation engineering systems, which was of great practical significance to the development of energy, transportation, environment, and medical and other fields. In order to make industrial robots adapt to more scenarios and easier to use, this situation is changing. For example, a safe and flexible cooperative robot has appeared, overcoming the shortcomings of traditional robots and making human–machine cooperation smoother.

**Fig. 3.**
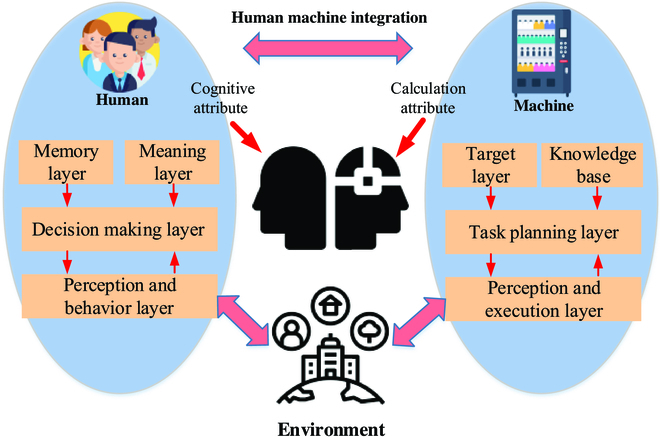
Architecture of human–machine integration (inspired by [125]).

Ramanathan [59] contended that the Industrial IoT (IIoT) in the era of 5IR was a new network infrastructure for the interconnection of people, machines, and things. IIoT would promote the formation of a brand-new industrial production, manufacturing, and service system and support the transformation and upgrading of the industrial economy. Figure 3 summarizes the architecture of human–machine integration.

As shown in Fig. 3, under the background of 5IR, human beings in the architecture of human–machine integration analyze and perceive the external environment through acquired perfect cognitive ability. Their cognitive process forms intentional thinking through the processing of memory layer, intention layer, decision-making layer, and perception and behavior layer. The machine perceives and analyzes the external environment through detection data. Its cognitive process depends on the target layer knowledge base, task planning layer, and perception and execution layer, forming formalized thinking.

Wang et al. [60] proposed that the 3 key technologies behind human–machine integration were AI, sensors, and HCI. Of these, AI was the brain of the robot and the core of human–machine integration. Deep learning and reinforcement learning in AI have been well applied. The widespread use of AI in the manufacturing industry has significantly impacted the manufacturing industry. Intelligent machinery, equipment, and new management concepts have been promoted [61]. For a mature manufacturing mode, the personnel must master the cutting-edge technology, and various production equipment must be designed with excellent quality. Embedding AI into the manufacturing process enables the robot to be more intelligent and flexible and operate autonomously in more complex situations. Sensing technology is an integral part of intelligent manufacturing. Intelligent sensors enable robots to imitate human sensory systems to sense the environment and promote the development of human–machine integration [62]. Intelligent devices and robots can realize seeing, listening, smelling, or feeling by combining relevant software to understand their environment and make corresponding responses intuitively [63,64].

The above research works suggest that the development of HCI is the only way to realize the integration of human and computer. In the context of 5IR, interactive technologies, such as graphic interaction, visualization technology, animation technology, speech recognition, natural language understanding, image and video recognition, motion capture, and virtual reality (VR), have all given birth to an important direction for the development of robots. In other words, strengthening human–machine interaction is an integral part of the development of 5IR.

### VRM in 5IR

Interdisciplinary and complexity require systematic approaches, such as systematic information physical systems. This systematic approach must model the interactions of various scales of dynamically interacting systems [65,66]. Modeling is challenging from all angles. It involves various systems, scales, data, and path dependencies generated by different industries and research disciplines [67]. It should be emphasized that 5IR also includes sectors other than manufacturing, such as life sciences, healthcare, agriculture, food, or energy. In addition, the participation of government, consumers, and society is the key to the acceptance of 5IR. In particular, biotransformation may lead to a new value but may not be related to the term industry. Integrating AI into the research and design process and the needs of different research disciplines in complex systems is necessary. The interrelationship and causality between multiple variables must be understood as a dynamic network that is constantly changing.

Abdel-Basset et al. [68] pointed out that with the continuous advancement of intelligent transformation and information construction of factories, large-scale simple production line enterprises deployed and applied various information systems and automation systems. This made the workshop management more important, and the requirements for the safety, availability, operation, and maintenance management of various equipment in the workshop got lower. Liu et al. [69] showed that the traditional monitoring system and equipment could not meet the needs of enterprises. Decentralized monitoring and integrated management were imperative for the factory. It could not realize the high-definition display, detail view, and other operations of the whole process of information status of the factory, equipment, production, and operation. Nowadays, manufacturing enterprises are also aware of the problems in production monitoring management. In other words, the lack of integrated planning management and decentralized monitoring has brought more challenges.

In the past technological revolution, robots and automation have incurred paradigm changes in global manufacturing [70,71]. 5IR aims to combine these cognitive computing skills with intelligent human thinking in collaborative operations. Therefore, it is conceivable that 5IR will bring about a change in norms and a fundamental change in industrial and manufacturing methods. Brown [72] observed that 3D digital visualization technology could collect, monitor, and analyze the real-time information of industrial equipment. These data involved temperature, humidity, rotation speed, vibration, and switch. Under normal conditions, alarms could be classified and managed to launch the alarm prompt automatically launched and position the corresponding equipment of the 3D IoT virtual factory. Meanwhile, different audible warnings were accompanied so the supervisors could timely capture the operational risks. At the same time, it could guide fault handling through 3D dynamic mode. The analysis result was transmitted to the manager terminal to display to managers. Then, one 3D page contained multiple information visualized by the 3D IoT-3D digital simulation technology. On this basis, each maintenance personnel could manage multiple machines, open up the information island, and boost work efficiency.

Cohen and Macek [73] mentioned that the real scene was highly restored through 3D simulation technology and a 3D digital visualization model of intelligent factory buildings, workshop structures, facilities, and equipment. Then, it vividly and precisely displayed the organizational relationship of different facilities, equipment shapes, and production processes. The digital model also visualized the distribution and operation of facilities and equipment and enabled users to browse the entire industrial site on the computer. Users interacted just like being on the spot. Furthermore, the system bound the equipment 3D model with the basic data to position the equipment and query its information. Cheung et al. [74] found that the IIoT-based 3D digital visualization could manage the asset library in the intelligent equipment association using the 3D modeling technique. The 3D modeling was associated with the equipment data and production data, where the 2-dimensional graphics were combined with the 3D model. The 3D scene could review the relevant information of other equipment. In the 3D simulation world, the user could accurately check the equipment's structure, component composition, and technical parameters by interacting with machines. They could comprehensively understand and master the equipment structure and diagnose and treat equipment faults.

The high-end manufacturing enterprises have felt the value of VR technology in industrial digitization. Consequently, the VR-based digitalization mode matures [75–77]. VR digital factory has improved the operation and management mode in the traditional manufacturing industry, enabling enterprises' maximum benefits in production and operation at the lowest management cost. Zeqiri et al. [78] reasoned that combining VR, information technology, and IoT could accurately track each link and equipment on the production line in the digital factory. On this basis, the production data could be processed in real time and visually displayed.

To sum up, in addition to the current factory simulation, the functions of the VR digital factory will be further improved in the future. Thanks to AI technology and big data algorithms, industrial production is fully intelligent, minimizing human involvement and allowing factories to function without even human involvement. In the future, with the in-depth application of VR digital factory system, the HCI function will be continuously strengthened, and the new system with intelligent management as the core will replace the static computer operating environment in the past. In addition, the system interface has also changed from flat to 3D. Meanwhile, managers can intuitively see production scenes and various data information, making production management more convenient.

## Application Values of DT in 5IR VRM

### DT content architecture and key technologies

According to the National Aeronautics and Space Administration, DT aims to create virtual twin models of physical entities. Liu and Xu [79] proposed a 3-layer cyber-physical production system centered on cyber-physical machine tool to illustrate the vertical integration of DT systems in various intelligent systems at different levels and the field-level horizontal integration of manufacturing facilities and resources. Armendia et al. [80] explored the application status of DT in CPSs. Stavropoulos and Mourtzis [81] mapped out the main architectures and applications of the DT in Industry 4.0 along the lines of manufacturing systems, manufacturing processes, and robotics, automation, and VR in manufacturing. It turns out that DT enable industry to discover physical problems faster, predict outcomes more accurately, and ultimately build better products. The virtual object can simulate and analyze the physical entity and monitor the operation state of the physical entity following the real-time feedback information. Further, it improves the simulation analysis algorithm of the virtual entity according to the collected operation data to guide decisions for the subsequent operation and improvement of the physical entity. Khan et al. [82] studied that the DT network would become the new direction of network planning, operation, management, and operation in the future. It would reflect the core value in 4 aspects: topology and traffic holography, full life cycle management from equipment to networking, real-time closed-loop network control, and network risk and cost reduction.

Zheng et al. [83] researched that the DT-based model was a dynamic replica of the whole life cycle of an entity or logical object in the digital space. It could achieve high-fidelity digital representation, simulation tests, and object state and behavior prediction based on rich historical and real-time data and advanced algorithm models. Schimanski et al. [84] found that integrating DT and lean construction was the key to project success. The DT-based industrial construction had 2 production lines: digital and logistics. In establishing a virtual model, the behavior model must be parameterized to reflect the changes in material properties and mechanical properties in parameters. The rule model was also parameterized. From the perspective of the physical model, in the finite element analysis software, the possible parameter changes at the behavior and rule level were modularized. A dynamic DT library was established. Table 2 lists the application areas of DT in 5IR.

**Table 2.** Comparison of application fields of DT technology in 5IR.

Author	Thesis title	Core point	Application fields
Deng et al. [127]	A systematic review of a Digital Twins city: A new pattern of urban governance toward smart cities	DT was the dynamic presentation of a physical entity's past and current behaviors or processes in a digital form.	Smart city governance
Yi et al. [128]	Digital Twins-based smart assembly process design and application framework for complex products and its case study	DT was the digital model of physical products in virtual space. It contained product information from product conception to product delisting.	Intelligent manufacturing and product assembly
Wang and Luo [129]	A Digital Twins-based big data virtual and real fusion learning reference framework supported by IIoT toward smart manufacturing	DT simulated and integrated multidisciplinary, multiphysical-quantity, multiscale, and multiprobability processes. It utilized a physical model, sensor update, and operation historical information to map the object in virtual space and reflect its whole life cycle process.	Intelligent manufacturing and industrial Internet
Wei et al. [130]	Implementation strategy of physical entity for manufacturing system Digital Twins	DT was a digital model of a physical entity based on a sensor. It could simulate and display specific things in the world.	Tool life prediction in numerically controlled machine tools
Al-Ali et al. [131]	Digital Twins' conceptual model within the context of the Internet of things	DT presented physical objects in the virtual space digitally. It simulated their behavior characteristics in the real environment.	Intelligent cloud computing platform
Zhuang et al. [132]	The connotation of digital twin, and the construction and application method of shop-floor digital twin	DT constructed the virtual things in the virtual space and was the projection relationship between virtual twins and their real objects in the physical space. The twin models were similar in shape and behavior.	Intelligent manufacturing and information physics systems
Yun et al. [133]	A Novel Digital Twins Architecture with Similarity-Based Hybrid Modeling for Supporting Dependable Disaster Management Systems	DT was a dynamic replica of the whole life cycle of an entity or logical object in the digital space. It could realize high-fidelity digital representation, simulation tests, and object state and behavior prediction. It was based on rich historical and real-time data and modern algorithms.	Disaster monitoring and prediction system
Yang et al. [134]	Application status and the prospect of Digital Twins for on-orbit spacecraft	DT digitally established a multidimensional, multidisciplinary, and multiphysical dynamic virtual model of physical entities. It simulated and characterized the physical entities' attributes, behaviors, and rules in the real environment.	Aerospace industry

DT has a wide range of industrial application scenarios. Wu et al. [85] defined DT as an innovative application of integrating a series of technologies, such as perception, transmission, calculation, modeling, and simulation. Its architecture included physical, data, model, and functional layers. The physical layer was the objects in the physical world, divided into tangible objects such as the human body, objects, and physical space and intangible objects such as business processes. The data layer was the foundation of DT-based application, consisting of data acquisition, transmission, processing, and storage. The model layer was the core of DT-based applications, using the modeling and other technologies to represent the digital image of real objects. In contrast, the function layer was the direct value embodiment of the DT. It stored the simulation results and visual services and offered them to business system applications to meet the needs of various application scenarios.

The mapping relationship of DT is bidirectional. On the one hand, based on rich historical and real-time data and advanced algorithms and models, the state and behavior of physical objects can be reflected in the digital world. On the other hand, simulation tests and analysis and prediction in the digital world can provide a decision-making basis for the instruction issuance of entity objects and the further optimization of the production process. It greatly improves the efficiency of analysis and decision-making [86,87]. Ning and Jiang [88] used the laboratory scale network physical system platform based on industrial communication networks and physical sensors for verification and found that both data-driven and model-based technologies were considered to be able to capture stealth attacks and prevent them. DT can offer the foundation for enterprise strategic decision-making through the decision support system (DSS). The most practical application of a visual DSS is to help users establish DT in the real world.

Sousa et al. [89] used the existing massive amounts of data information and visualization techniques to model business decision-making. The model could evaluate the current state, diagnose past problems, predict future trends, and encourage more comprehensive and accurate decision-making for business operations. Thus, a perception prediction action-oriented intelligent DSS was formed. First, the intelligent DSS used sensor data or data from other systems to determine the current state of the target system. Second, the system modeled and predicted the possible results under various strategies. Finally, an analysis platform was employed to find the optimal strategy against the desired goals.

### 3D visualization modeling of digital factory's DT in 5IR

Modeling digitization is the process of digitizing the physical world. This process must express physical objects as digital models recognized by computers and networks [90,91]. Modeling can simplify and model human's understanding of the physical world or problems. The purpose or essence of DT is to exchange information for energy through digitization and modeling. It eliminates the uncertainty of various physical entities, especially complex systems, with less energy. Therefore, establishing a digital model or information modeling technology of a physical entity is the source and core technology of creating DT. They are also the core of the digitization stage.

Wu et al. [92] proposed that constructing the DT model involved a conceptual model and model implementation method. Of these, the conceptual model described the architecture of the DT system from a macro perspective and presented considerable generalization. Model implementation methods, such as modeling language and model development tools, focused on realizing the model technically. The related technical methods and tools showed a diversified development trend. So far, DT-oriented modeling languages like AutomationML, UML, SysML, and XML have been developed [93–94]. While some use general modeling tools like computer-aided design, more models are based on special-purpose tools such as FlexSim and Qfsm.

Various conceptual models have been proposed by scholars, which are listed as follows. (a) The micro-kernel DT platform architecture based on the simulation database provides support for correcting simulation models and more realistic projection through the active management of real-time sensor data by the simulation database [95]. (b) DT-oriented automatic model generation and online simulation. The static simulation model is selected as the initial model. The dynamic simulation model is automatically generated from the static model based on the data matching method, and the simulation accuracy is improved by combining multiple models. Finally, the online simulation is realized through real-time data feedback. (c) The conceptual framework of the DT modeling process includes physical entity, data layer, information processing, and optimization layer. On this basis, the twin model construction is guided by industrial production. (d) The DT modeling based on model fusion builds complex virtual entities by combining multiple mathematical simulation models. It proposes a virtual entity calibration method based on anchor points [96]. (e) The implementation framework of full-parameter DT divides the DT into 3 layers: physical layer, information processing layer, and virtual layer. It realizes the upper-layer DT application based on the data acquisition, transmission, processing, and matching. (f) The DT 5-dimensional model composed of physical entities, virtual entities, connections, twin data, and services emphasizes the driving effect of twin data. The twin data are collected from physical data, virtual data, service data, and knowledge of physical devices, virtual devices, and services. The application ideas and schemes are discussed for the DT 5-dimensional model in many fields [97,98]. (g) According to the data acquisition to application, the DT model is divided into the data assurance layer, modeling and calculation layer, DT function layer, and immersive-experience layer. Successively, each layer realizes data acquisition, transmission and processing, simulation modeling, function design, and result presentation.

**Fig. 4.**
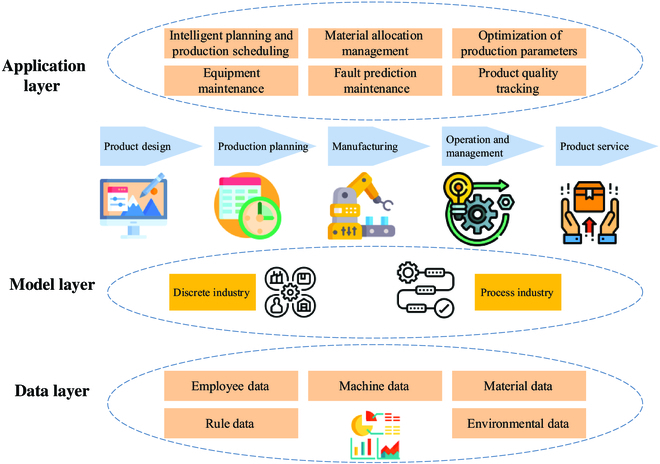
DT plus whole process management of industrial production and manufacturing (inspired by [126]).

Sun et al. [99] believed that there are 2 differences between DT and digital factories in the process industry. First, the focus was different. The DT focused on the industrial production line and did not involve too many enterprise management levels. Second, there were different functions. The process industry had many complex physical and chemical reactions in production. The construction of its DT involved digital 3D modeling and must carry out mechanism or data-driven modeling for the process industry. Through the full dimension fitting of the physical entity, the function of independent operation could be achieved to provide a more appropriate solution for the process industry. Figure 4 presents DT plus whole process management of industrial production and manufacturing.

As shown in Fig. 4, in the 5IR context, the application of DT to the whole process management of industrial manufacturing mainly includes the data layer, the model layer, and the application layer. In the data layer, the data of employees, machines, materials, rules, and environment are collected using multiprotocol and edge computing technologies. In the model layer, the whole life cycle of industrial manufacturing is optimized and managed, including product design, production rules, manufacturing, operation management, and product service processes. In the application layer, the whole life cycle of industrial manufacturing is intelligent scheduling, fault maintenance, and quality tracking. The application layer is mainly responsible for intelligent scheduling, fault maintenance, and quality tracking for the whole life management cycle of industrial manufacturing.

The professional modeling software outputs the 3D model and uses Photoshop and other drawing software to make maps. The lighting information is baked into the texture through the renderer. Then, the general model format and the baked map are output through the model software to complete the 3D model. The completed 3D model and map are imported into the visualization platform for debugging. The lighting and environment details are simulated in the visualization platform according to the equipment site environment. It makes animation and embeds a 2-dimensional data board to enrich the scene details and complete the important step of DT creation.

Padovano et al. [100] pointed out that in DT, virtual twin modeling was faced with slow speed, low degree of reduction, high threshold, low efficiency, and great difficulty in real-time visual development driven by physical world data. Catalano et al. [101] divided the rapid 3D modeling technology into 2 types. One was the 3D modeling based on tilt photography, which applied to constructing large-scale environmental models. The other was the reverse modeling based on the 3D laser point cloud data, which applied to the reverse modeling of single buildings and equipment. The core steps of the 3D reconstruction method based on tilt photography could be summarized as image acquisition, multiview image adjustment, multiview image dense matching, and texture mapping. Combined with the tilt photography 3D modeling, point cloud, and Unity real-time 3D rendering technology, a virtual DT system was proposed using the Unity 3D platform. The platform completed the real-time data driving and displayed it in Unity 3D. It realized the rapid modeling of the virtual twin scene and the visual display driven by the real-time data of the physical world [102–104].

Through the analysis of the abovementioned scholars' research, it is found that DT shows significant advantages in the development of 3D models of smart factories and 3D models of smart city parks. DT enables intelligent factory 3D models and smart city park 3D models. Intelligent 3D equipment is a 3D visualization DT-enabled smart factory built based on 3D modeling and 3D visualization. It uses modern information technologies, such as physical networks and cloud computing [105,106]. 3D visual modeling, 3D model, and 3D VR technology are used to build a unified organization and management coordination framework, business management platform, and external service platform. The platform provides innovative management and operation services for industrial equipment managers.

### Modeling method of DT platform based on industrial data

Recently, extensive research is on the theory and methods of data mining, generating various data mining tools. However, these tools have not successfully processed large-scale multidimensional datasets. Therefore, people use visualization technology in data mining and rich visualization methods to express multidimensional data intuitively. Then, the unique cognitive ability of humans is used to guide the mining process.

Therefore, a new direction has emerged in the industrial big data-oriented visual analysis field: visual data mining. Galletta et al. [107] used visualization techniques to establish a good communication channel between users and data mining systems. The research enabled users to use their rich industry knowledge to regulate and constrain the mining process and improve its results. It broke the black box mode of traditional mining algorithms and improved users' trust in the mining system. In visual data mining technology, the direct interaction ability of visualization was the key to the mining process. The research on the application form and use method of visualization technology in data mining were urgent for visualization [108,109]. From the perspective of technical application, physical modeling is inaccurate in complex system modeling. More new-generation AI algorithms will be combined with numerical control machine tools to open up new technical routes. Thereby, the stability and accuracy of prediction are improved to make the machine tools have better knowledge learning, accumulation, and application abilities.

The essence of DT is to use virtual twin modeling to restore the physical world scene. The traditional modeling technology is slow and low in restoration, while the data-driven real-time visualization of the physical world has a high threshold, low efficiency, and great difficulty. By comparison, the rapid 3D modeling technology can easily help model virtual twin scenes and display physical world data [110,111]. Industrial 3D modeling data visualization-based intelligent monitoring system provides excellent innovation and development environment through intelligent infrastructure and service system. It focuses on the industrial visual modeling model. Intelligent industrial data visualization-based intelligent monitoring system can quickly, accurately, and effectively conduct chemical product debugging and information processing. Cao et al. [112] deducted that the industrial intelligent monitoring system would provide various data visualization support for industrial factories in real time. It improved the production efficiency of industrial industries and formed an effective aggregation of intelligent industrial data visualization with industry as the core.

DT multiview image dense matching can extend the classical least square matching algorithm to multiview images to form collinear conditions. This method is based on the constraint of the geometric relationship of the core line. It searches the 1-dimensional space on the core line image and combines the gray observation equation to calculate the object square and pixel coordinates. Texture information of the DT model is one key indicator affecting the display effect in the later stage. The traditional method uses the camera photos for uniform light and orthogonalization. Then, it pastes the processed texture images on the surface of the 3D model by mapping.

The 3D laser scanning system works like this. It uses the transmitter to emit laser pulse signals to the target object. The pulse signals are reflected on the object's surface, and the laser receiver on the instrument receives the DT. Finally, the distance between the target object's surface and the scanner is calculated according to the transmission and reception time difference for the pulse signals and the propagation speed of the pulse signals. The distance between the scanner and the object surface measured by the scanner and the angle information in the horizontal and vertical directions can accurately record the object surface's spatial data [113–115]. Then, the high-precision point cloud data can be computed. Finally, the reverse 3D model can be reconstructed using the processed point cloud data.

The project implementation contents and steps can be determined as per the DT project's functional requirements of the digital chemical factories. The whole DT platform framework is divided into workshop DT, equipment management and operation, and maintenance monitoring center [116,117]. (a) The DT platform of the workshop is established. The basic application model and data content are built by the DT digital chemical factory through the modeling platform. The digital model of the DT platform is established. The factory-level application's data integration and basic functions are built through the DT platform. Thereby, the virtual simulation of the DT platform at the factory and equipment levels is completed. Then, it maps the production line of the physical entity into the DT model. Then, the status of each piece of equipment on the production line is controlled in real time. (b) Construction of equipment management system. On the basis of the DT platform of the workshop, the life cycle account of the whole factory-level production equipment is established through the mapping of the Data Twins. With early warning and intelligent notification, relevant personnel can be notified through tasks for maintenance. (c) Construction of operation and maintenance monitoring center. It induction production data board, video monitoring, quality detection, personnel monitoring, and positioning systems. The production data board can visualize the production statistics in real time. Video monitoring provides a panorama of the plant area and real-time images of key monitoring areas. The quality inspection makes comprehensive statistics on such indicators as product abnormality, comprehensive yield, product line yield, single process qualification rate, and process defect history.

### Enabling technology of DT applied in 5IR

DT have been more widely spread in recent years. Meantime, thanks to the development of the IoT, big data, cloud computing, AI, and other new-generation information technologies, the implementation of DT has become possible. AI can automatically perform data preparation, analysis, fusion, and deep knowledge mining of twin data without the participation of data experts through the intelligent matching optimal algorithm. As such, it generates various types of services. With the support of AI technology, DT can significantly improve the value of data, responsiveness, and accuracy of various services and empower various vertical industries.

The DT ecosystem can be divided into the basic support layer, data interaction layer, model construction and simulation analysis layer, common application layer, and industry application layer. AI technology is mainly applied in the simulation analysis layer. In the simulation analysis layer, how to realize value extraction through efficient mining methods in large-scale data is one of the key problems of DT.

DT-oriented information analysis technology realizes intelligent information analysis and auxiliary decision-making through AI intelligent computing models and algorithms and advanced visualization technology; it also realizes monitoring and visualization of physical entity operation indicators, automatic operation of model algorithms, and online preview of physical entity future development to optimize physical entity operation. DT was first applied in industrial manufacturing and perfectly connected the physical and information worlds. Chen et al. [118] reviewed state-of-the-art process analytical technology development, process modeling approaches, and data integration research. The authors concluded that DT is a key technology driving the shift toward agile and intelligent manufacturing. Psarommatis and May [117] analyzed the literature on the adoption of a systems approach to zero-defect manufacturing DT and is guided by preliminary findings on the lack of structured and standardized digital twin application development methods. This culminates in a systematic and critical analysis of the answers to some fundamental questions in the context of the zero-defect manufacturing DT, thus contributing to the knowledge. With the continuous development of big data, IoT, and AI technologies, the form and concept of DT are constantly expanding and gradually upgraded to multidimensional and dynamic management models and solutions, which also profoundly impact retail, education, media, and other fields.

The practical significance of the AI-oriented DT business plan is also reflected in the store operation. The store management can simulate, verify, and predict the overall life cycle of the physical store with the help of historical data, real-time data, and algorithm. As such, it can improve the store management's sensitivity to market trend changes and assist the management in making scientific decisions in the supply chain. Especially, the logistics field is very suitable for applying DT technology for scientific research and innovation. Moshood et al. [120] reviewed the deployment issues and technologies that support DT in an attempt to assess how DT can be used to improve the visibility of logistics supply networks. It was found that DT will help companies to develop predictive metrics, diagnostics, forecasting, and physical asset descriptions for their logistics and can overcome the challenges of implementing DT in the logistics industry. Leal et al. [121] proposed an ontology for interoperability assessment to provide a basis for the application of DT in manufacturing in the context of 5IR. DT enables intelligent logistics systems. That is to say, it imitates human intelligence, forming the ability of thinking, perception, learning, reasoning, and judgment and solving some problems in logistics. The potential advantage of DT lies in testing various hypothetical situations or establishing a training and testing environment. It fuses AI and big data to test the operation of the logistics system in advance.

Under the framework of traditional supply chain management theory, both the optimal order quantity model and the optimal replenishment lead time model are based on the decision-making of manufacturers, dealers, and retailers to maximize their utilization. The "bullwhip effect" in the supply chain can be eliminated through comprehensive supply planning, collaborative planning, forecasting, and replenishment [122,123]. However, there is still a zero-sum game phenomenon in which the profits of the whole supply chain are different. These problems will be solved in the twin of the digital supply chain. Defraeye et al. [124] considered a DT-empowered supply chain as a DT system in the supply chain. It combines prerule techniques (time series and machine learning), decision tools (e.g., AI and operations research optimization), and digital word generation techniques to form a digital twin-based DS. The DT-enabled supply chain can break through the traditional supply chain's response speed and cost bottleneck, effectively connecting the upstream and downstream. Also, it carries out exemplary management and intelligent decision based on data movement, improves the efficiency of supply, and reduce the cost of supply.

## Discussion and Prospect

### Discussion

Here, X has 2 meanings. On the one hand, X represents intersection. With the advancement of transformation, production and consumption need to be integrated in a certain sense. On the other hand, X also represents the future and the unknown. New technologies, including BDT, AI, blockchain technology, and quantum computing, emerge in endlessly. The future will be full of infinite possibilities. With the continuous advancement of factory automation and information construction, deploying and applying various automation systems and information systems make workshop management as a product production more important. The workload also increases, the requirements for collaborative work are higher, and the requirements for safety, availability, operation, and maintenance management become higher. On the other hand, with the continuous evolution of the intelligent construction of the factory, it will face more challenges due to the lack of unified planning and other constraints: the large scale of the factory, scattered deployment of intelligent monitoring equipment, high inspection pressure, high labor cost, and inability to find problems in time.

The current factory monitoring lacks a centralized and intuitive visual monitoring management platform. The management personnel cannot understand the on-site production situation and process flow in a real-time, comprehensive, and accurate manner. Building a 3D visual DT factory platform is imperative on the basis of the above problems. The solution of the DT factory is to realize the overall management by integrating 3D visualization technology, rapid modeling technology, real-time state monitoring technology, and camera monitoring technology. The DT factory platform integrates the 3D high-precision model of the workshop, process flow, equipment attributes, real-time data of equipment, and plant operation management data. It can intuitively display the process flow of the production workshop, remotely control and manage workshop production, and improve operation management efficiency. At the same time, it provides customers with complete and high-value-added product solutions to realize intelligent and fine management of enterprises. In Industry X.0 era, DT can be virtual prototypes at the design stage, which can be adjusted through simulation experiments. Then, it invests in purchasing physical prototypes. In addition, designers can quickly simulate the operating conditions by verifying the virtual products and completing the virtual design and operation. After service encapsulation, model services can be used through service search, matching, scheduling, and invocation.

Likewise, a review and analysis of research conducted by scholars in related fields in recent years reveals a range of effects that DT brings about through its combination with industrial manufacturing enterprises. On the design side, DT provides the means to achieve the integration of information technology and manufacturing and helps companies move at high speed toward the path of digital transformation by digitally creating highly realistic virtual models of physical objects and simulating, analyzing, and predicting their behavior. On the production side, employees who leverage DT innovation will have the ability to expand their engagement with online devices and complete work, various business process improvements with great continuity and accuracy. DT can validate product or system design expectations prior to deployment, improve factory productivity, optimize product performance or maintenance yards, and integrate complex manufacturing processes to close the loop on product design, manufacturing, and intelligent service. This shows that DT will become one of the key technologies for the future development of digital industry.

### Development challenges of industrial DT

Under the policy wave of new infrastructure, the traditional manufacturing industry is eager to transform, profit, and expand the market through IIoT technology. Two major challenges are faced in building the DT technology scene of the factory.

First, from the perspective of traditional computer-aided design 3D modeling and VR technology, the modeling workload is large, and the cycle is long. It is not easy to expand. At present, the 3D laser scanning system is recommended. The 3D laser scanning system integrates the laser scanner, digital camera, software, and auxiliary equipment. It can obtain the 3D point cloud data and texture (image) data of the target object in a noncontact and rapid manner and build a true 3D digital model of the scanned object through data processing and 3D modeling. There are commercial 3D laser scanning systems abroad, but the price is relatively high. Domestic suppliers also provide 3D laser scanning modeling services.

Second, data analysis in the factory has a wide range of dimensions, including performance, capacity, energy consumption, quality, cost, and efficiency. Each dimension involves many links, and data collection and modeling are difficult. In the paint spraying quality analysis of the robot arm, the parameters include the paint manufacturer, the paint mixture ratio, the ambient temperature, and humidity. Additionally, it also covers the moving speed of the robot arm, the nozzle pressure, and the flow rate. These data are in different links. For example, the paint mixture ratio depends on the experience of workers and cannot be accurately quantified, making the modeling and analysis process very difficult. The prediction and analysis of product quality and production line capacity involve even complicated factors. They are closely related to enterprise products and business. Therefore, the factory DT construction is a long-term, continuous exploration and accumulation process, and there is no shortcut. The factory DT also apply to the process industry, such as steel, petrochemical, food, and beverage. The slightly different information is based on different industry characteristics.

The third is the construction of the DT network system. Under the massive network data, data modeling should not only ensure the rich functions of the model but also take into account the flexibility and extensibility of the model. Consequently, building an efficient and hierarchical basic and functional model is more complicated. In the case of high real-time demand, model simulation and verification on the DT network will lead to the extension of the system running time. Therefore, different processing mechanisms must be added in different network application scenarios. At the same time, real-time requirements will further improve software and hardware performance requirements. In addition, the communication network usually has many elements, a wide coverage, and a long service time. Therefore, DT are bound to be a huge and complex system. Its collection, storage, model design, and application will become more complex. The requirements for the software and hardware of the system will become higher.

## Conclusion

This review comprehensively analyzes the application of DT in the 5IR context. It is found that 5IR complements and extends the signature features of Industry 4.0. It highlights aspects of the determinants that place industry in the European society of the future. These are of an economic or technological nature and have important environmental and social dimensions. In terms of technology, 5IR wants to capture the promise of advanced digitization, big data, and AI, emphasizing the role these technologies can play in meeting new and urgent needs in the industrial, social, and environmental landscape. This means using data and AI in production to successively increase production flexibility and make the value chain more robust. A review and analysis of the literature related to the application of DT in VRM in 5IR reveals that DT is a key technology in industrial manufacturing. It can create value, reduce time-to-market, optimize plant equipment and finished product performance, and provide insight that is unmatched by any other solution. At the same time, integrating DT should be the next inevitable step for global manufacturers as the cost decreases, the number of suppliers increases, and the availability of the advanced technologies that make up the DT increases. Thereby, DT is superior to traditional data charts for 3D visualization and synchronized information transmission and monitoring interaction technology. It is gradually being recognized by industrial enterprises to help them step into the era of intelligence. The subsequent study will prospect the potential application value of DT in 5IR and the subsequent potential value to provide research directions for the subsequent development of intelligence in the industrial field and industrial manufacturing in the era of Industry X.0.
